# ResNet-32 and FastAI for diagnoses of ductal carcinoma from 2D tissue slides

**DOI:** 10.1038/s41598-022-25089-2

**Published:** 2022-12-02

**Authors:** S. Phani Praveen, Parvathaneni Naga Srinivasu, Jana Shafi, Marcin Wozniak, Muhammad Fazal Ijaz

**Affiliations:** 1 Department of Computer Science and Engineering, Prasad V Potluri Siddhartha Institute of Technology, Vijayawada, 520007 India; 2 Department of Computer Science, College of Arts and Science, Prince Sattam bin Abdul Aziz University, Wadi Ad-Dawasir, 11991 Saudi Arabia; 3 Faculty of Applied Mathematics, Silesian University of Technology, 44-100 Gliwice, Poland; 4 Department of Mechanical Engineering, Faculty of Engineering and Information Technology, The University of Melbourne, Grattam Street, Parkville, VIC 3010 Australia

**Keywords:** Breast cancer, Breast cancer

## Abstract

Carcinoma is a primary source of morbidity in women globally, with metastatic disease accounting for most deaths. Its early discovery and diagnosis may significantly increase the odds of survival. Breast cancer imaging is critical for early identification, clinical staging, management choices, and treatment planning. In the current study, the FastAI technology is used with the ResNet-32 model to precisely identify ductal carcinoma. ResNet-32 is having few layers comparted to majority of its counterparts with almost identical performance. FastAI offers a rapid approximation toward the outcome for deep learning models via GPU acceleration and a faster callback mechanism, which would result in faster execution of the model with lesser code and yield better precision in classifying the tissue slides. Residual Network (ResNet) is proven to handle the vanishing gradient and effective feature learning better. Integration of two computationally efficient technologies has yielded a precision accuracy with reasonable computational efforts. The proposed model has shown considerable efficiency in the evaluating parameters like sensitivity, specificity, accuracy, and F1 Score against the other dominantly used deep learning models. These insights have shown that the proposed approach might assist practitioners in analyzing Breast Cancer (BC) cases appropriately, perhaps saving future complications and death. Clinical and pathological analysis and predictive accuracy have been improved with digital image processing.

## Introduction

Globally, ductal carcinoma is the second most significant reason for cancer-related mortality observed in women and the most frequent kind of cancer in both men and women. Breast cancer affects about 250,000 women in America each year, killing over 46,000. Breast cancer mortality rates have dropped by approximately 40% in the last two decades. This is an incredible feat made possible by advances in early diagnosis and more precise treatment options^1^. Over 95% of women with early-stage breast cancer survive 5 years or longer. Early identification and treatment of breast cancer are related to better outcomes. Still, it is also associated with a lower level of care, implying less aggressive treatment and minor invasive surgery than later detection and treatment. As a result, each woman must have the best opportunity to diagnose breast cancer in its earliest stages. 7.8 million women have been treated for and survived breast cancer over the past 5 years. In women of any age, breast cancer may arise. Breast cancer is no exception to the importance of early detection. Spotting breast cancer at a preliminary stage reduces the risk of premature mortality^2^.

**Figure 1:**
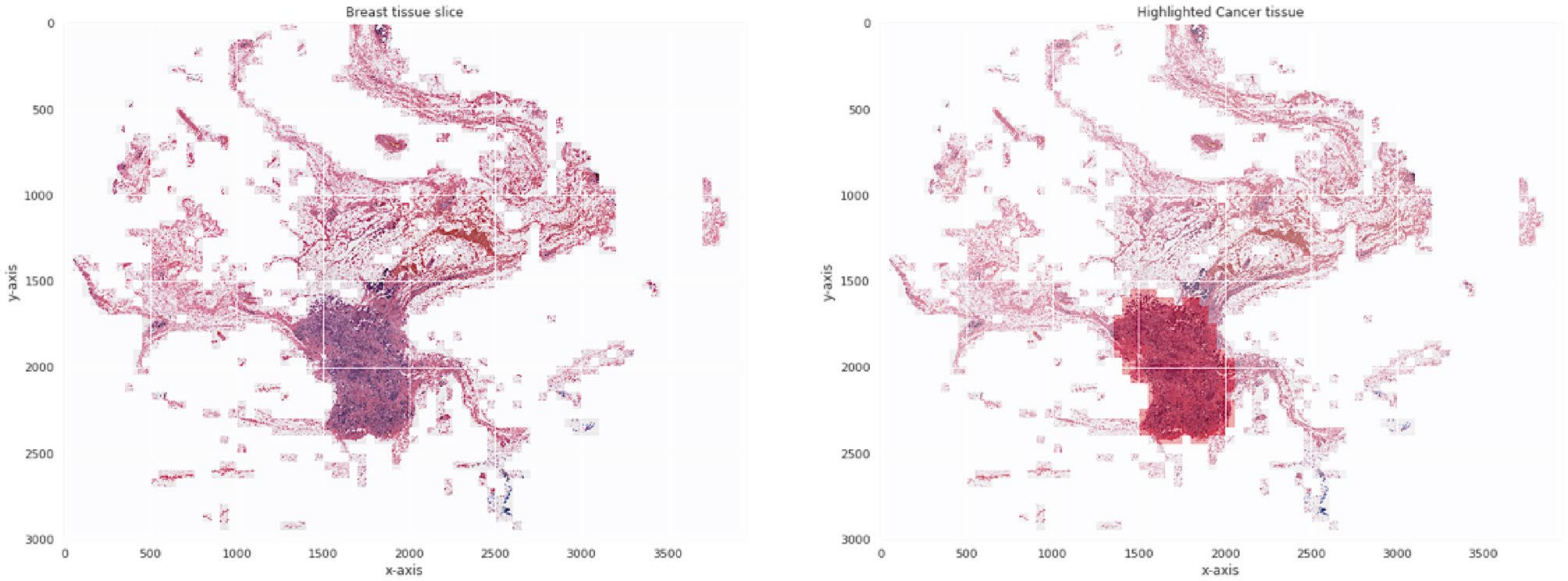
Image representing highlighted abnormal tissue region.

As a result of aberrant cells being found inside the breast milk duct, ductal carcinoma occurs. Breast cancer in its initial stage is known as ductal carcinoma. According to cancer statistics, the gap between the number of people diagnosed with cancer and those who survive is widening daily. Consequently, prior detection of breast cancer is now a primary priority for many women^3^. The imaging technologies like mammograms, ultrasound, Computer Tomography (CT), digital breast tomosynthesis, and Magnetic Resonance Imaging (MRI) are the most regularly utilized imaging modalities. Breast cancer is often diagnosed by palpation and frequent radiography or ultrasound imaging check-ups. A breast tissue biopsy is performed if the check-up exam reveals the risk of malignant tissue development. Pathologists may analyze the microstructures and components of the tissue through breast tissue samples. Histology allows for the differentiation of healthy and non-and malignant tissues and a prognosis assessment. Breast cancer imaging utilizes nuclear medicine imaging methods to identify and classify axillary lymph nodes and stage the disease at a distance. Tumor categorization and identification in mammography using computer-aided diagnosis (CAD) systems. Radiation therapists highly advocate this technique for spotting early signs of illness and malignant breast cancers^4^. Figure 1 presents the smart diagnosis model output that highlights the breast tissue affection region, Where the image on the left is the normal image given as input for the CAD model, and the image on the right shows the highlighted region of the suspected tumor. The reports of CAD on assessing the patient for ductal carcinoma were applied to perform surgeries, radiation therapy, hormonal therapy, immunotherapy, chemotherapy, and surgical planning. Hence, it is desired to have a preliminary assessment models for better treatment.

Some studies identify tumour size and location, while others focus on acquisition techniques and breast quadrants. During the past few decades, scientific research has focused on prototyping the Deep Learning models concerned with diagnosing breast cancer using thermography. Deep learning can automatically extract characteristics from a dataset it is being trained on. The deep learning models strive to acquire high-level characteristics from input in an iterative manner. This reduces the necessity for domain knowledge and hardcore feature extraction^5^.

The current study is motivated by the scope of research that would assist in precisely identifying the abnormality in the early stages of cancer. Breast cancer growth is not identical for all cases, and each demands a unique diagnostic strategy. Early identification of breast cancer may enhance clinical results and allow more aggressive types to be managed clinically. Researchers and doctors have been fascinated by the idea of non-invasive early cancer diagnosis for decades. Improved diagnostic technology has made it possible to identify even the smallest lesions in breast cancer, allowing for the creation of very successful screening programs. Hence, there is a demand for a robust mechanism to diagnose the abnormality^6^. The deep learning models like ResNet-32 are robust in analyzing the input MRI images and deliberating the results accurately. The objectives of the current study are as follows.

- Data acquisition and localizing of the region of interest from the available tissue slides of the ductal carcinoma-affected persons.
- To design a deep learning model for learning insights from the input data and precisely identifying the image's tumors.
- Using the FastAI technology with ResNet-32 would assist in faster convergence with reasonable computational efforts for processing the input.
- Analyzing the efficiency of the diagnosis model across various accuracy evaluation techniques like sensitivity, specificity, F1 Score, and Multi-fold cross-validation.
- Statistical analysis of the performances with other cutting-edge models was used for a similar study.

The overall paper is arranged as the “Introduction” section, which presents a brief outline of the field of study, motivation, and objectives. Section “Literature review” presents the literature of the study, and section “Materials and methods” offers the methods and dataset description used in the current study. Section “Proposed ResNet-32 over FastAI” presents the feature engineering and FastAI with the ResNet-32 model. The next section presents the “Results and discussion”, and the final section offers the conclusion and future scope of the study.

## Literature review

Some various approaches and techniques are widely used in the classification of the tissue slides, which include the conventional image segmentation techniques like K-Means and classification models like support vector machine (SVM), Logistic Regression (LR), K Nearest Neighbour (KNN), Random Forest (RF) and Decision Tree (DT) used in the process of classifying the slides. And various deep learning models like Artificial Neural Network (ANN), Convolutional Neural Networks (CNN), Recurrent Neural Network (RNN), and multiple models like VGG-16, Inception V3, AlexNet, DenseNet-169 are being used in the diagnosis of ductal carcinoma. The current section discusses the different models used in evaluations and their limitations.

K-Means Clustering (KMC)^7^ technique is used to assess breast cancer cases, and the study has stated that the model has obtained a precision of 0.857. And the K-Means algorithm generally needs lesser computational efforts than its counterparts as the model doesn’t need any training. In the same study, the K-means algorithm was used with the Self-Organized Maps (SOM), and the hybrid algorithm exhibited a precision of 0.921 from the experimental outcome. The model has converged with a minimal number of iterations on performing the hybridization compared to SOM alone. However, the model lacks the non-linearity in identifying the divergent breast cancer cases. The other unsupervised techniques are based on clustering, namely the Fuzzy C-Means (FCM) algorithm^8^. The performance of FCM is comparatively better than the K-means algorithm in terms of accuracy. However, the FCM technique needs more computational efforts over multiple iterations. The experimental results of FCM for segmentation of breast cancer have obtained 86%. In the FCM approach, there is a possibility of over-segmenting the images, resulting in misleading results.

Breast cancer diagnosis and classification using image mining experimented by Mohanty et al.^9^, has obtained a classification accuracy was 97.7% because of the Gray-Level Co-occurrence Matrix (GLCM) and intensity characteristics. The large dimensions of the matrix and the good correlation of the Haralick characteristics are limitations of the GLCM technique. An empirical study on BC detection using an SVM classifier-based learning approach minimizes diagnostic variance and boosts diagnosis accuracy to combat the restriction of individual model performance. The model has exhibited an accuracy of 97.68%, which is reasonably fair compared to other conventional approaches. The cutting-edge classification algorithms have few limitations in dealing with complex real-time problems. For example, the SVM approach is generally poorly suited to large datasets. Using SVM when the data set includes more noise, such as when the target classes overlap, may not always be the best option.

On the other hand, algorithms like RF perform well for small-size data sets, and they need tremendous training and are more susceptible to overfitting the model. Similarly, the other predominantly known classification algorithms like Multi-layer Perceptron (MLP), which works through backpropagation, have yielded better performance. The downside is that the overall number of parameters might quickly become excessive. Because of the redundancy at such large dimensions, this is inefficient. Another drawback is that it ignores spatial information. The performances of various cutting-edge classification models are presented in Table 1.

**Table 1:** Performances of various classification models used for BC diagnosis.

Classification approach	Accuracy (%)
LR^10^	66.3
KNN^10^	66.1
SVM Model^11^	89.1
K-NN^11^	85.2
RF^11^	82.4
ANN^11^	86.27
C 4.5 Decision Tree^12^	93.47
Naive Bayes^12^	95.93
Supervised fuzzy clustering^12^	95.57
Weighted voted-based ensemble^13^	97.42
Multilayer perceptron neural networks^14^	98.0

Using deep learning techniques, medical image analysis may be improved by extracting features and increasing efficiency. Convolutional neural networks are used in deep learning, a machine learning technique (CNN). Unlike the other feature extraction methods, images may be extracted from a dataset using the CNN approach. Features may be extracted from various picture sections using convolution in this method. It is observed in various experimental outcomes the CNN model has exhibited a reasonable performance. In one of the experimental analyses, the CNN obtained an accuracy of 96.15% over the BreakHis dataset^15^. A Fuzzy Neural Network (FNN) technique^10^ using an enhanced Gini index and a random forest-based feature significance measure method was developed for the early detection of BC. The study on FNN has claimed accuracy of 99.3% over the limited-size dataset with 32 features. The RNN-based tumor prediction model^16^ experimented over the Wisconsin dataset with an accuracy of 93.86%. But the RNN models have the limitations of vanishing gradients and complex training procedures. Other robust DL models have exhibited better performance in classifying the tumors, including the AlexNet, DenseNet, ResNet, MobileNet, VGG16, etc.

Advanced models like AlexNet are divergently used in Computer-Aided Diagnosis (CAD) to predict diseases like Alzheimer’s, diabetes, cardiovascular diseases (CVD), COVID-19, and many other diseases. The experimental analysis for breast cancer identification has observed accuracy of 93.8% over the BreakHis dataset for 100 epochs^17,18^. DenseNet, which obtained excellent performance on image classification challenges, was expanded to address the issue of semantic segmentation. DenseNet uses feature reuse even further by employing skip connections to assist the up-sampling route in recovering geographically extensive data from the deconvolutional path^19,20^. The study on DenseNet with the Squeeze-and-Excitation (SENet) module has exhibited an accuracy of 89.5% over the BreakHis dataset^21,22^. The other studies on breast cancer prediction based on the Visual Geometry Group (VGG-16) have obtained an accuracy of 86.2% over the BreakHis dataset^23^. MobileNet V1 and MobileNet V2 are the models that are tremendously used in the classification of various classification algorithms in healthcare^24^. The models have exhibited 86.7% and 85.2% accuracy using breast cancer prediction^25^. Various deep learning models are used to assess the abnormalities from the Biomedical imaging technology to identify and classify the tumors. The accuracies of each of those models are presented in Table 2.

**Table 2:** Accuracies of various classification techniques used for breast cancer diagnosis.

Deep learning approach	Accuracy (%)
CNN^15^	96.15
CNN^26^	87.0
ResNet-18^22^	93.3
GoogleNet^22^	79.3
AlexNet^17^	93.8
VGG-16^23^	86.2
AlexNet^18^	94.6
DenseNet^21^	89.5
VGG-19^21^	84.3
MobileNet V1^25^	86.8
MobileNet V2^25^	85.2

## Materials and methods

The current study uses a deep learning model to identify the ductal carcinoma cases from the patient’s tissue slides. The ResNet-50 model is used with the FastAI framework for faster convergence and optimization of GPU-Optimization. On the other hand, ResNet-32 handles issues like the vanishing gradient, and many network layers may be trained without increasing the error rate. The current section discusses the FastAI and Linear Binary Pattern (LBP) for feature selection using the ResNet-50 model to classify the cancerous tissue slides.

### FastAI

**Figure 2:**
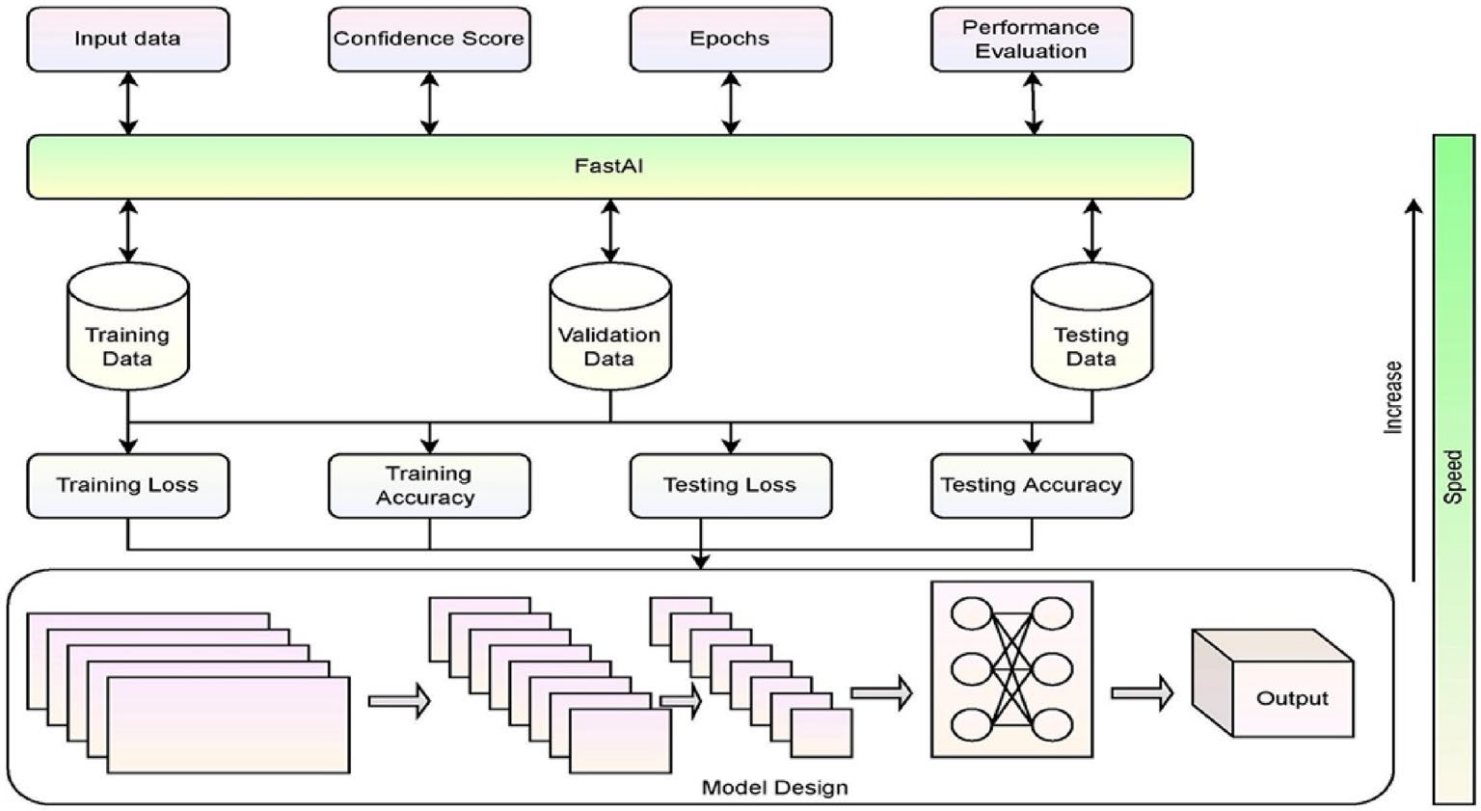
FastAI framework for deep learning model.

FastAI^27^ is a comprehensive deep learning package that provides specialists with various libraries and packages, offering speedy and precise outcomes in deep learning^28^. Furthermore, it gives researchers low-level characteristics that may be mixed and combined to create novel modeling techniques. FastAI is the first DL component that provides users with a trusted platform for all of the most commonly used DL technologies for time-sensitive series, pattern matching, content-based filtering, and statistical analysis. FastAI is built around three key design goals: quick productivity, easy configuration, and a flexible framework. It works by a series of lower-level APIs that serve as the foundation for FastAI. As a result, a client who wants to redefine sections of the high-level interfaces with particular criteria to meet their needs to acquire how to utilize the lower-level APIs. FastAI is based on two key design goals: being approachable and productive with flexible. The architecture of the FastAI model in classification is presented in Fig. 2.

### Local binary pattern

A Local Binary Pattern (LBP) is an image texture information that works with the thresholds of the surrounding pixels depending on the magnitude of the intensity of the current pixel^29^. LBP technique readily captures local spatial distributions and greyscale variance in a picture. LBP is frequently employed in various image processing applications for feature identification and image enhancement. It is calculated to encode pixel intensity differences in the neighborhood region. Images must be of adequate quality for making the accurate predictions from the data^30–32^. The LBP value for the centre pixel must be calculated. We may begin at any nearby pixel and make clockwise or counterclockwise, but the ordering has to be consistent across all pixels in the picture and images in the collection. With a 3 × 3 neighbourhood, the 8 neighbours to conduct a binary analysis. The binary test results are saved in an 8-bit vector, which we subsequently transform to decimal. The image does not need to do any external padding to be performed for processing. The size of the kernel used for processing is of size 3 × 3 with 9 elements for processing. LBP for any given image was processed using a set of steps, which were discussed below.

- For any given image *img*, the *pix*(*x*, *y*) with *n* neighboring pixels in a radius of *r*$.$
- Calculate the intensities between the corresponding pixel *pix*(*x*, *y*) and all the *n* neighboring pixels. The difference evaluation is shown in Eq. (1)
  1 $$Dif = \sum\limits_{i = 0}^{n - 1} {f(g_{c} - g_{i} )2^{n} }$$
- Threshold the intensity values ensuring that all negative values are denoted as 0, and all positive values are allocated 1, creating a bit vector.
- Substitute its intensity value at (*x*, *y*) with the decimal number corresponding to the P-bit vector.

Because of its intrinsic efficiency and robustness, the LBP technique is often used to construct texture features enabling the classification of image pixels^33^. The adaptive threshold generates the Binary Vector^34,35^. The threshold *T* is asses as shown in Eq. (2)

2 $$T = a \times \left[ {1 + s\left( {\frac{k}{R} - 1} \right)} \right]$$

**Figure 3:**
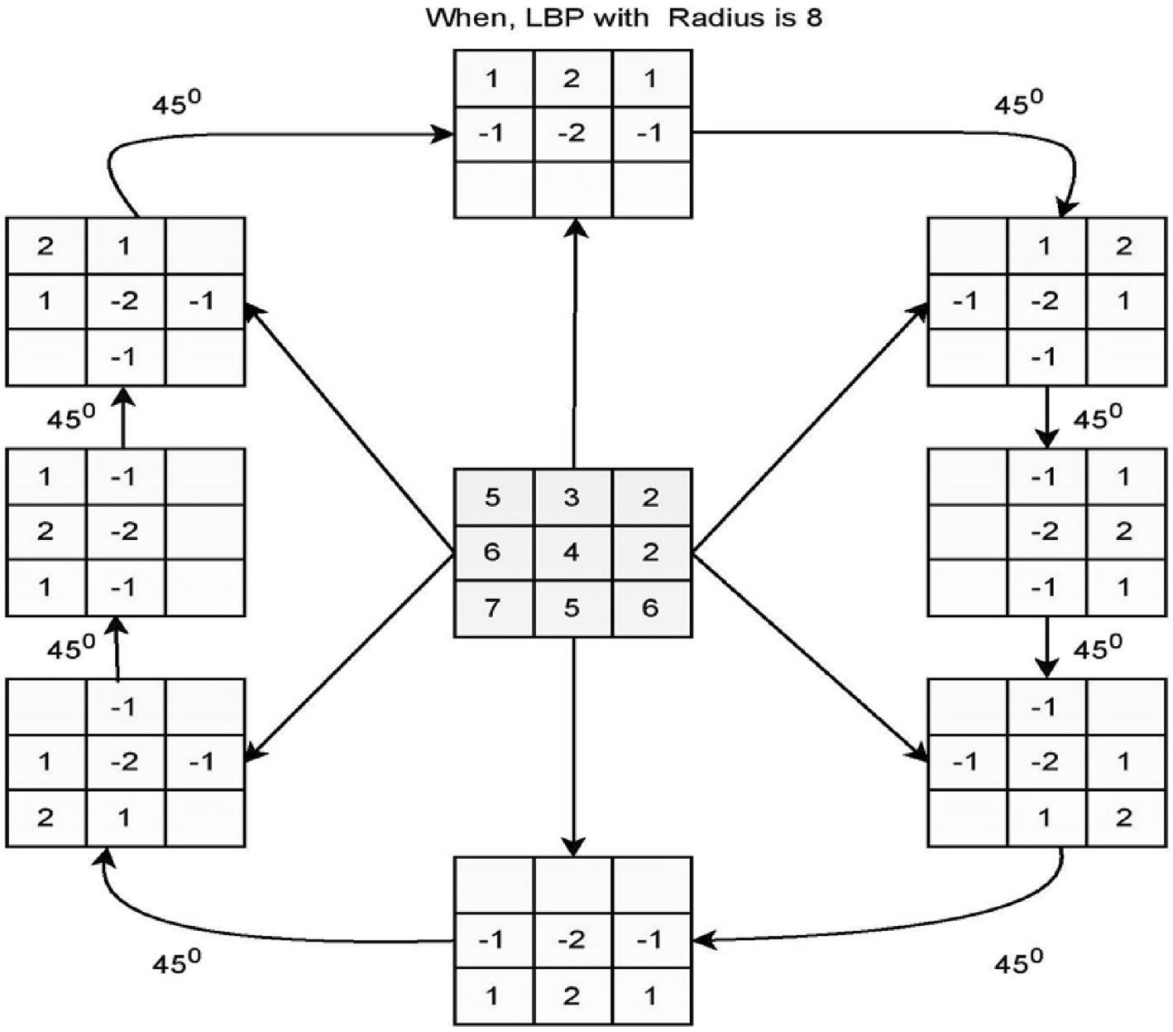
Diagram representing the LBP operations.

From Eq. (2), the variable *a* denotes the local average of the pixel intensities, *k* designates the standard deviation of the pixel within the region, and variable *s* is the user-defined parameter whose values are always positive. The variable *R* denotes the dynamic range of deviation. The LBP with a radius of 8, is shown in Fig. 3.

### Feature score and approximation of the relevance score

The feature score assessment is crucial in identifying feature engineering^36,37^. In the considered image *img*, where the texture of the images is designated as the dimension *C* of the feature vector. The pixel information of the image is represented using the *m* × *n* matrix over *p* features. The variable $img_{i}^{k}$ designates the *k*th feature value where *k* = 1, 2…*p*. Generally, the *k*th The feature is assumed as the middle feature for convivence. The matrix of the image is shown as Eq. (3).

3 $$img = \left[ {\begin{array}{*{20}l} {img_{1,1}^{1} } & \cdots & {img_{1,k}^{k} } & \cdots & {img_{1,n}^{p} } \\ {} & {} & \vdots & {} & {} \\ {img_{k,1}^{1} } & \cdots & {img_{k,k}^{k} } & \cdots & {img_{k,n}^{p} } \\ {} & {} & \vdots & {} & {} \\ {img_{m,1}^{1} } & \cdots & {img_{m,k}^{k} } & \cdots & {img_{m,n}^{p} } \\ \end{array} } \right] = \left[ {f^{1} \ldots f^{k} \ldots f^{p} } \right]$$

From Eq. (2), the variables $({img}_{{1,1}}^{1}\dots {img}_{1,k}^{k}\dots {img}_{1,n}^{p})\in {\mathbb{R}}^{m}$ represents the *n* rows represent the texture and the variables $({img}_{1,n}^{1}\dots {img}_{k,n}^{k}\dots {img}_{m,n}^{p})\in {\mathbb{R}}^{n}$ represents the columns represent the texture information. To assess the significance and the relevance of each feature $f$ for the tissue classification, the score $s$ is calculated as shown in Eq. (4)

4 $$s = \frac{1}{t}\sum\limits_{i = 0}^{t - 1} {\left( {img_{i}^{k} - \mu^{p} } \right)^{2} }$$

In Eq. (3), the variable $\mu^{p}$ denotes the mean of pixel intensities of all images associated with feature *p*. The value of $\mu^{p}$ is assessed through Eq. (5)

5 $$\mu^{p} = \frac{1}{t}\sum\limits_{i = 1}^{t} {img_{i}^{p} }$$

The features are ordered in the highest to the lowest pattern of *s* to choose the most appropriate feature, recognizing that the feature with the largest difference is the discriminant.

### Feature score updation

The feature score values are being updated over the epochs, and the measures of inter-class variance, intra-class variance, and entropy are being considered for updating the feature score^38^. The latest feature score is being identified using the variable *s*′ with the entropy $\varepsilon$ is shown in Eq. (6).

6 $$s^{\prime} = s \times \frac{{\alpha_{v }^{(k)} }}{{\beta_{v }^{(k)} }} + \frac{1}{\varepsilon }$$

From the above equation, the variable ${\alpha }_{v}^{(k)}$ designates the inter-class variance concerning to the feature *k* and ${\beta }_{v}^{(k)}$ designates the intra-class variance concerning feature *k*. The inter-class and intra-class variance is being assessed using Eqs. (7) and (8) respectively.

7 $$\alpha_{v}^{(k)} = \sum\limits_{c = 1}^{n} {\frac{{p_{c} }}{p}\left( {m_{c}^{(k)} - m^{(k)} } \right)}^{2}$$

8 $$\beta_{v}^{(k)} = \frac{1}{p}\sum\limits_{c = 1}^{n} {\sum\limits_{{q = \omega_{c} }} {\left( {z^{(k)} - m_{c}^{(k)} } \right)} }^{2}$$

From the above equations, the variable *p* designates the sum of samples and *p*_*c*_ designates the sum of samples that falls under the class *c*$.$ The variable ${m}_{c}^{(k)}$ designates the mean of the values over the samples in class *c* concerning to the feature *k*. *m*^(*k*)^ designates the mean of all sample values towards feature *k* across all classes. The variable* z*^(*k*)^ denotes the value of *z*th sample concerning to feature *k*. The variable *ω*_*c*_ denotes the associated weight of the sample concerning to class *c*. One way to quantify the level of randomness included in an image is by calculating its entropy, also known as its average information. Entropy is a metric used for assessing the uncertainty of the image used in processing. When all the intensities in co-occurrence matrix *M* are equal, the image has the most uncertainties resulting in high entropy value. The distribution of gray levels in the image has become quite convoluted at this point. The formula for assessing the entropy is presented in Eq. (9).

9 $$\varepsilon = - \sum\limits_{x} {\sum\limits_{y} {M(x,y)\log (M(x,y))} }$$

### Image transformation and scaling

**Figure 4:**
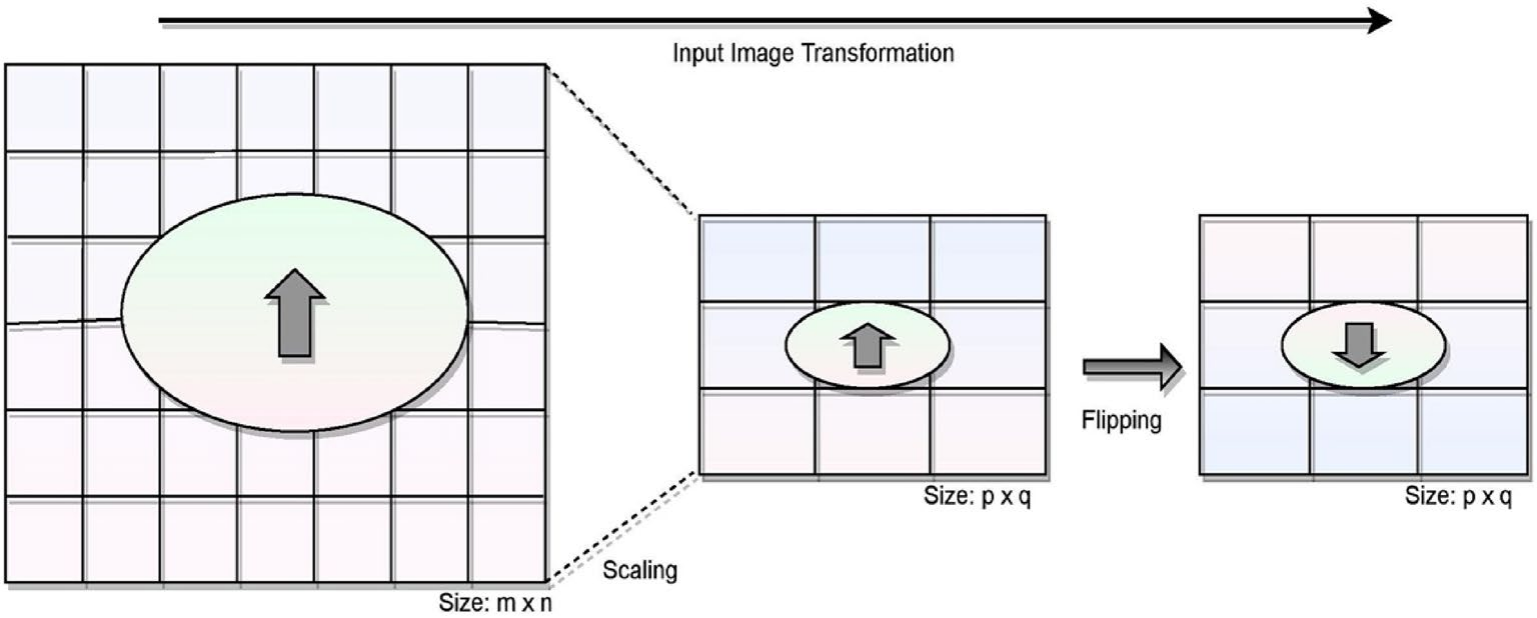
Image presenting the transformation on the input image.

The input tissue slides of ductal carcinoma are pre-processed concerning the scaling and the transformations towards flipping the images for further processing. The original images of size 50 × 50 are scaled down to 32 × 32 × 3 for further processing. There are some cases where the scaling of the images would impact the significant features for classification. Still, the images are scaled down for a faster and more convenient way of processing and faster computations of the images. The images were easily handled over multiple layers of the deep learning model on scaling the image. Figure 4 presents the operations performed as part of image transformation.

**Figure 5:**
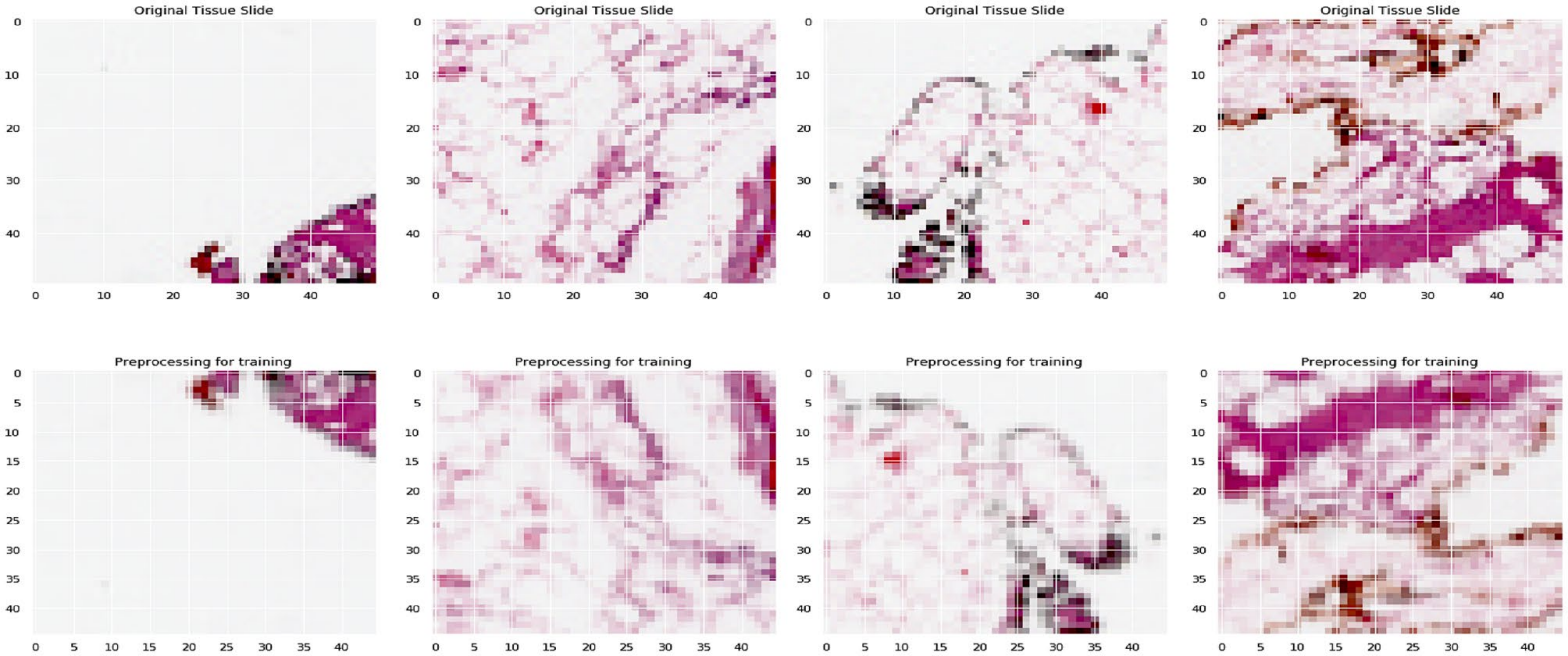
Processed image for training purposes.

The images are flipped horizontally and vertically when scaling down the original images to the desired size for further processing. From the above figure, the original image of size *m* × *n* i.e., 50 × 50, which is scaled down to the size *p* × *q* i.e., 32 × 32 for further processing. The image scale would assist in faster training with small size image compared to the one that are larger. As each pixel region over the tissue slice may be rotated, we significantly improve the data's diversity. They will not miss the spatial connections among pixels of the images, and it is lesser significant that some nearby image pixels are flipped in different orientations. Flipping the images will be on the vertical axis in horizontal flip, and the flipping will be on the horizontal axis in vertical flip. The flipping operations would allow the model to learn the insights from the input data over multiple angels that can build a robust model. The outcome of the image pre-processing is shown in Fig. 5. The processed images are used in training the ResNet-32 model.

### Dataset description

**Figure 6:**
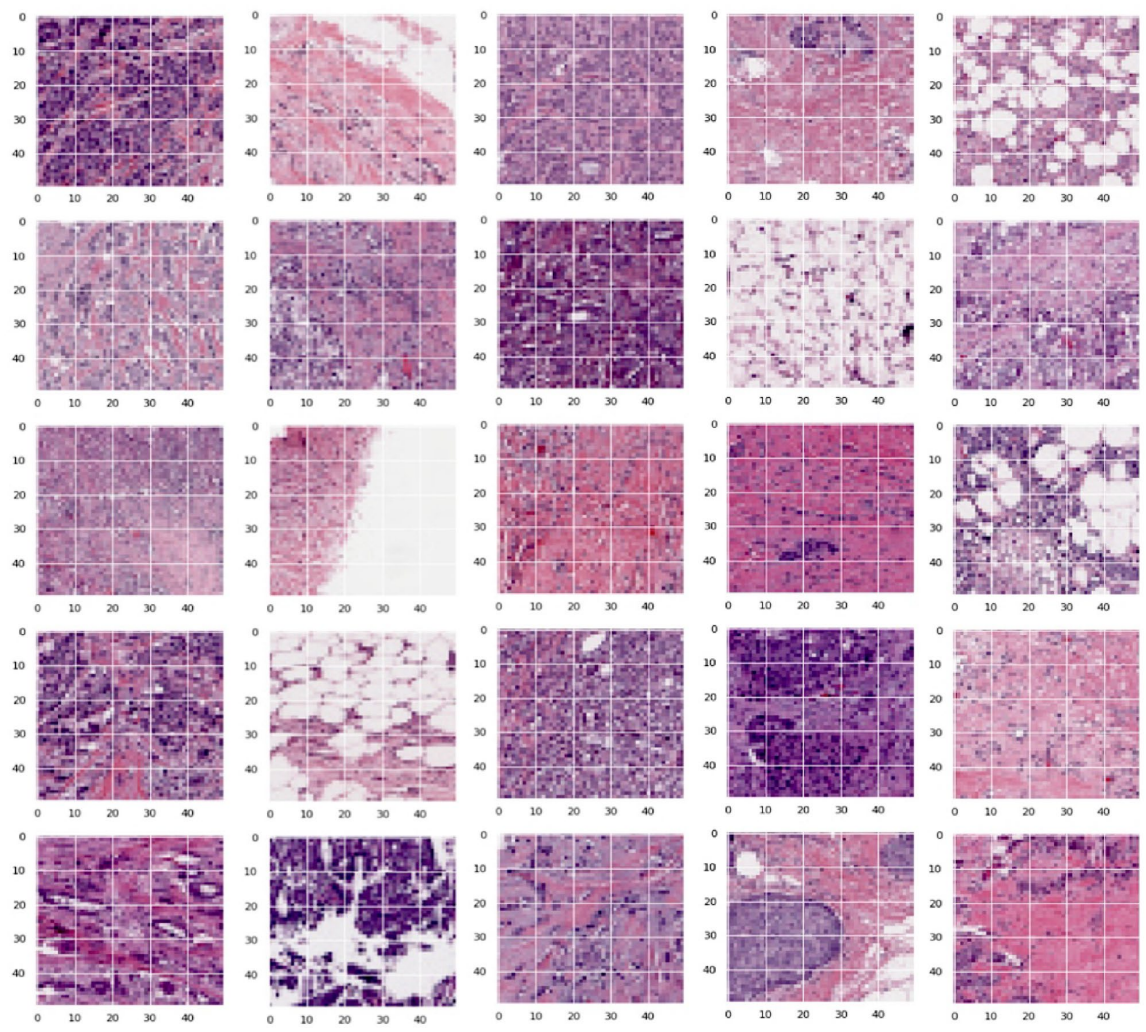
Sample images are part of the IDC dataset.

The dataset is open-source, which is used in the current study is Invasive Ductal Carcinoma (IDC), that are part of Breast Histopathology Images^39,40^ that comprised 162 whole mounted slides images of size 50 × 50 of breast tissues samples processed at 40 ×. The whole-mount slides of IDC negative and IDC positive breast cancer specimens are included in the collection. The dataset consists of 198,738 IDC negative and 78,786 IDC positive. Every image is assigned with either 0 or 1. Where, 0 is for the images that are IDC negative, and 1 is for that IDC positive. Figure 6 presents the images in the dataset.

### Implementation platform and used libraries

This experiment is conducted using Kaggle's compiler in the online platform. The FastAI package over the PyTorch frameworks for transfer learning is used to create the in-depth learning approach mentioned in this paper. The ResNet-50 model was created using the Python programming language. Various libraries are used in the implementation, including sklearn, torch, torchvision, glob, matplotlin, and Numpy. The execution interface is implemented in Microsoft Windows 11 64-bit environment over the Inter^®^ Core i7-9750H device.

## Proposed ResNet-32 over FastAI

**Figure 7:**
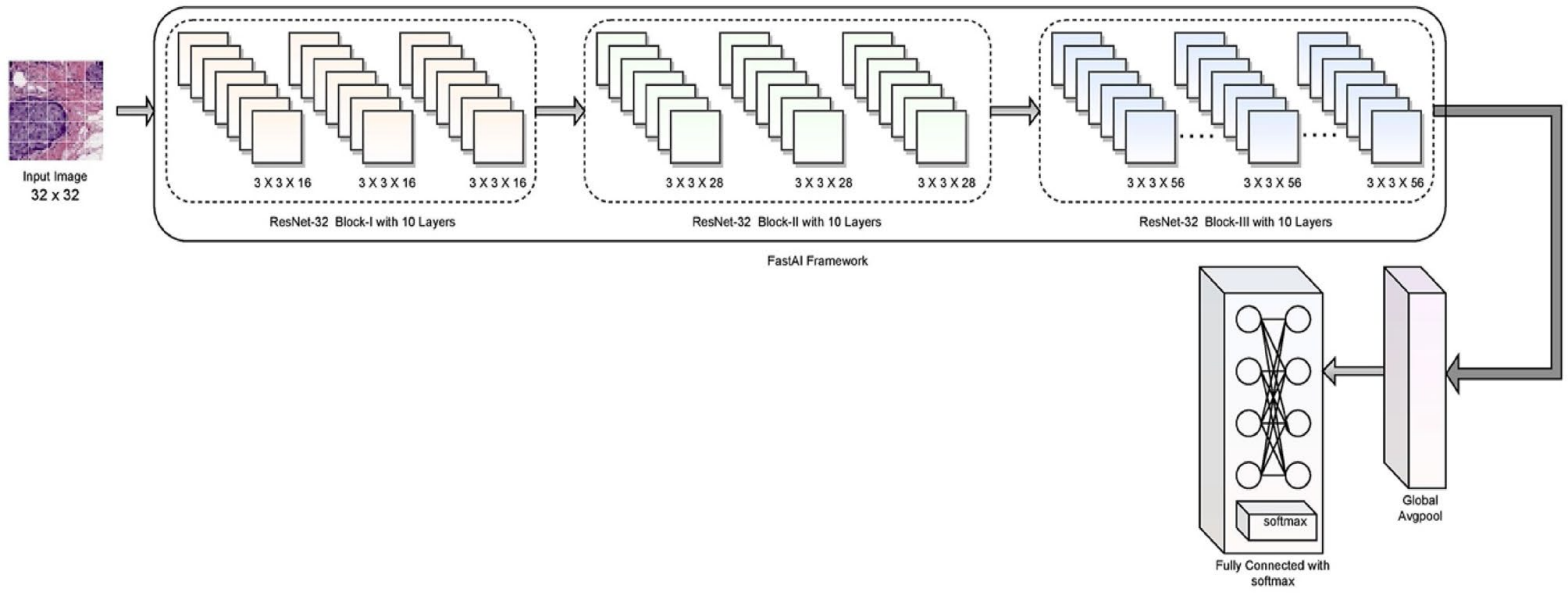
Presents the layered architecture of ResNet-32 for classification.

Residual Neural Network (ResNet-32) is a 32-layer CNN comprising 30 Convolutional layers (Conv_Ly) alongside a MaxPool and a fully connected layer with softmax layers. ResNet is a kind of Deep Learning Model that builds a network by stacking residual connections on top of each other. Even when the architecture becomes more complex, the ResNets model remains as efficient as ever, making it a better option than alternative architectural models. ResNet-32 model exhibits identical performance like other versions of ResNet architectures, despite of having few layer than its counterparts. As each of the residual blocks in ResNet would result in two independent pathways, ResNet architecture with *r* residual blocks would possess 2^*r*^ possible pathways for processing the data. As a result, Minimizing the number of layer in the archicture does not have significant impact on the performance of the model. Moreover, working will fewer number of layers would result in faster computations and the capability of training networks is improved. Figure 7 represents the layered approach of the ResNet 32 model.

The ResNet architecture relies on the CNN model concerning the layers part of classification architecture. The network comprises layers like the convolutional, maxpool, fully connected, and softmax layers^41^. The model is comprised of 30 convolutional layers that are arranged across three batches of variable input and output size. The following are the functions of each layer in classifying the IDC images. The input images are of size 32 × 32 in dimension across three channels. The details of each of those layers that are part of the proposed architecture are discussed in the present section of the study, and the size of kernels and stride of the network are shown in Table 3.

- The convolutional layer adds filtering over the input image, which activates the neurons. Whenever the filtering is repeatedly performed over the input, The activation function transforms the input in a more non-linear manner, allowing it to learn the insights from the data and accomplish increasingly difficult classification tasks. The outcome is a feature vector denoting the intensity associated with the detected features at various input points organized in a non-linear fashion. After creating a feature map with numerous filters, it may be passed via activation functions like Rectified Linear Unit (ReLU). A convolutional layer's filter is much smaller than the original image, and the process executed among these two individuals is often a dot product. Equation (10) defines the generic form of a non-linear activation function, with weight specified by the variable $\omega$ and bias over an input $v$ represented by the variable $\beta$.
  10 $$C = f\left( {\omega \times v + \beta } \right)$$
  ReLU is the non-linear pairwise activation function used with the proposed model's convolutional layer and efficiently handles the vanishing gradient. When it deals with an input whose value is negative, it is confined to a value of 0. Otherwise, it is positive. It is unrestricted. This combination of either of these would result in building a better regularisation classification model. Regularization results in a sparse representation, which allows for efficient and robust inference and training. The equation for the ReLU is shown in Eq. (11) for input *x*.
  11 $$f(x) = maximum(0,\;x)$$
- Global Average Pooling (GAvP) is similar to the Squeeze-and-Excitation component. It uses a big pooling kernel with a long stride (to save some work) and one 1 × 1 convolution in the module. G layers are used to minimize a three-dimensional tensor's spatial dimensions. GAvP layers, on the other hand, conduct a more intense dimensionality reduction, in which a tensor with dimensions *x* × *y* × *d* is reduced in size to dimensions 1 × 1 × *d*. GAvP layers use the mean of all *x* × *y* values to simplify each *xy* feature map to a single integer.
- The fully connected and softmax layers are combined as a single layer in the ResNet-32. A convolutional network's fully connected layers are a multilayer perceptron that seeks to map the ${k}_{1}^{i-1} \times {k}_{2}^{i-1} \times {k}_{3}^{i-1}$ activating neurons from the combination of preceding layers into a probability distribution. Thus, the multilayer perceptron's output layer will contain ${k}_{1}^{i-x}$ output neurons, where *x* specifies the layers as in multilayer perceptron. The equation for the fully connected layer is presented through Eq. (12)
  12 $$fc_{x}^{i} = \sum\limits_{p = 1}^{{k_{1}^{i - 1} }} {\sum\limits_{q = 1}^{{k_{2}^{i - 1} }} {\sum\limits_{r = 1}^{{k_{3}^{i - 1} }} {\omega_{x,p,q,r}^{i} \times (M_{x}^{i - 1} )_{q,r} } } }$$

**Table 3:** Presents the kernel size associated with the ResNet-32 architecture.

Layer	Kernel	Stride	Activation Function
Input	32 × 32 × 3		
Batch 1: Conv_Ly	3 × 3 × 16	2	ReLu
Conv_Ly	1 × 1 × 16		
Conv_Ly	3 × 3 × 16		
Batch 2: Conv_Ly	3 × 3 × 28	2	ReLu
Conv_Ly	1 × 1 × 28		
Conv_Ly	3 × 3 × 28		
Batch 3: Conv_Ly	3 × 3 × 56	2	ReLu
Conv_Ly	1 × 1 × 56		
Conv_Ly	3 × 3 × 56		
Global Maxpool	3 × 3		
Fully Connected Layer	1000-d		1 × 1 × 64
Output	2		

From Eq. (12), the component ${\omega }_{x, p, q, r}^{i}$ denotes the weight parameter associated with the activation maps of the convolutional, non-linearity, rectification, and pooling layers. The softmax layer is a common non-linear activation component used in deep learning models. It is a function that presents the probabilities associated with the class of classification problem. The mathematical model for softmax probability *S*^42^ assessment is presented in Eq. (13)

13 $$S = \frac{{e^{pj} }}{{\sum\nolimits_{x = 1}^{n} {e^{{p_{x} }} } }}$$

From Eq. (13), the variable *p* is the arbitrary vector over the valves [*j* = 1, 2, 3…*n*], where *n* is the size of the vector. Affine transformations of input features define the softmax regression model's outputs regardless of how non-linear it is. The details of the number of trainable parameters and the associated convolutional layers are presented in Table 4. As the input image size, the ResNet-32 model is limited, and the number of trainable parameters is less than the other versions of the ResNet^43^. The initialization parameters of the ResNet-32 are presented in Table 5.

**Table 4:** Details of network parameters used in the evaluation.

ResNet-32 architecture details	Values
Number of Layers	32
Number of Conv_Layers	31
Number of trainable parameters	0.46 M
Error rate	7.14

Parameter	Value
Initial learning rate	0.00001
Dropout ratio	0.3
Bias	0.01
Batch size	60
Number of classes	2
Number of epochs	30
Activation	ReLu
Number of layers	50
Random state	2
Stride	2

**Table 5:** Hyperparameter values of various models.

	Training accuracy	Training loss	Validation accuracy	Validation loss
CNN^44^	92.25	0.12	81.93	0.56
VGG-16^44^	99.75	0.01	79.00	0.29
ConcatNet^44^	95.90	0.11	86.23	0.43
Proposed model	95.34	0.12	94.83	0.32

Table 4 presents the initialization parameters used in the proposed network.

### Hyperparameters

**Figure 8:**
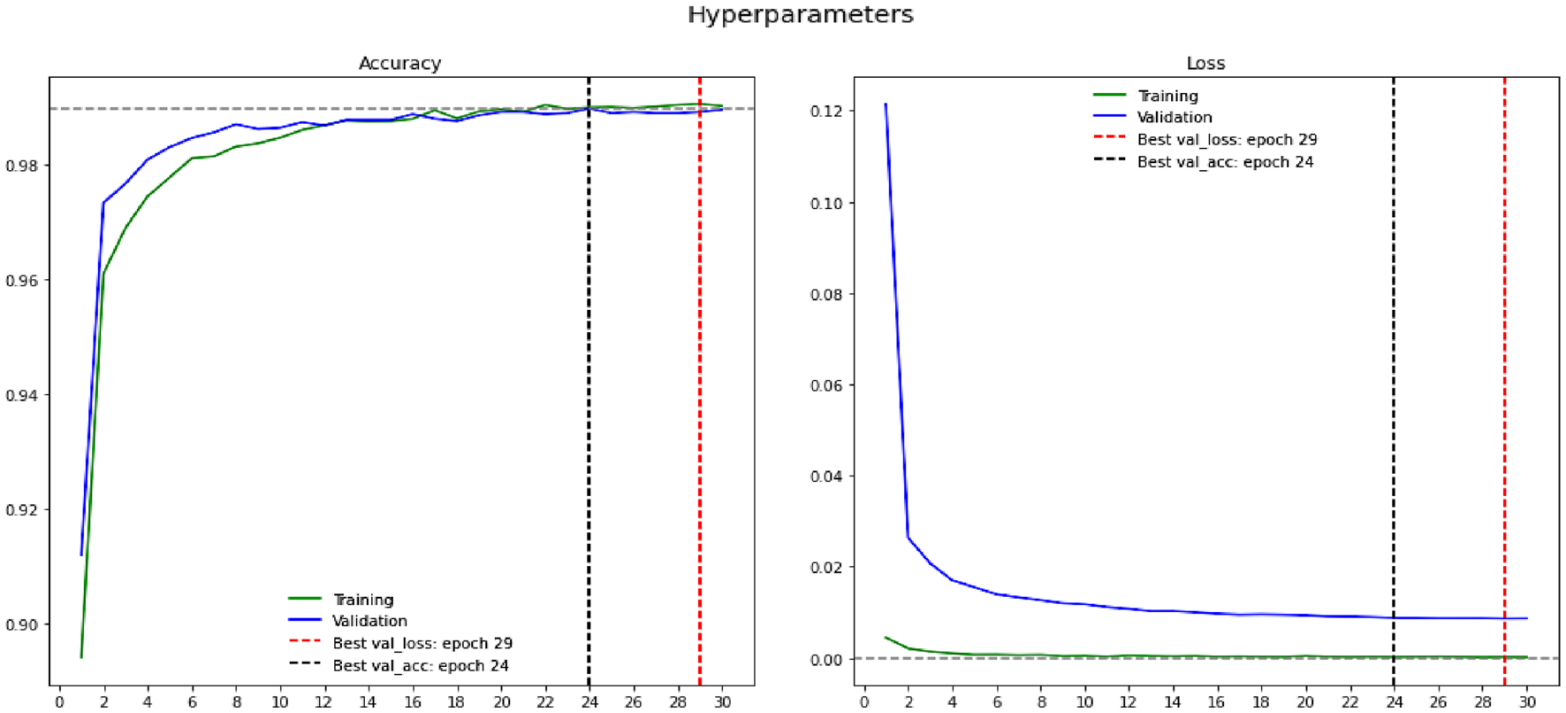
Graphs presenting the hyperparameters of the proposed model.

The hyperparameters of every deep learning model are exceptionally significant in determining the model's performance. Hyperparameters are variables that allow you to fine-tune the training process of the DL model. The hyperparameters include the training accuracy, training loss, validation accuracy, validation accuracy, Batch Size, Learning rate, and the epochs of training that assist in fine-tuning the model to attain optimal performance by handling the overfitting and underfitting scenarios^24^. For hyperparameter exploration, the objective is to determine the optimum possible configuration of hyperparameters. Unpredictability in the training data might impact the model's ability to precisely predict an appropriate outcome from validation data, known as overfitting. The training loss graph shrinks over time, as does the validation loss graph, until it stabilizes and begins to rise. Because it doesn't learn from the data or generalize well, a model is assumed to be underfitted. The accuracy and the loss measures associated with the proposed are shown in Fig. 8.

It can be observed from the accuracy graph of the proposed model, a good models would have the validation and training loss that declines to a stable point with a small difference among two final loss values. The best valuation loss is usually greater than the training loss and whose loss value is least among all the epochs. The validation loss is minimal at epoch 29, which is assumed to be the best validation loss. The validation accuracy is slightly lower than the training accuracy in most of the times, the best accuracy notes across the epochs assumed to be best validation accuracy, in the current study the best validation accuracy is recorded at epoch 26. The hyperparameters associated with various deep learning models used in classification over a similar area of study are presented in Table 5. It can be observed from the tabulated values, that the efficiency of the FastAI driven ResNet-32 model, which is reasonably better than other conventional models used in similar studies. The ResNet-32 network over the FastAI framework has improved validation accuracy.

**Figure 9:**
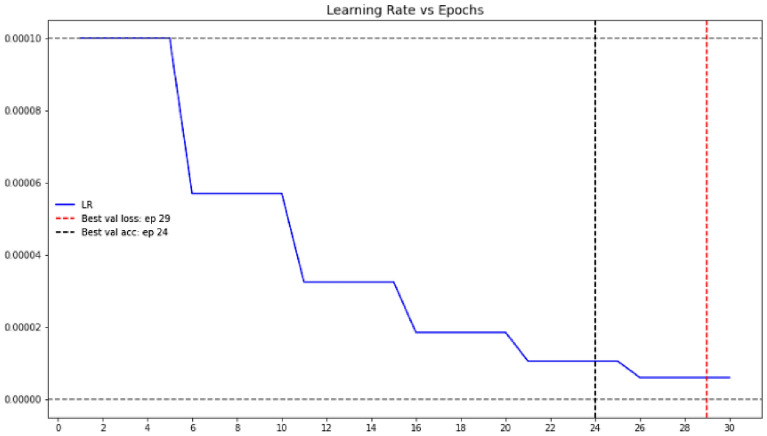
Learning rate graph of the ResNet-32 network.

Learning rate is a crucial factor in determining the model's performance. The learning rate is relatively low, and the training process will be comparably sluggish in updating the associated network weights. In contrast, a higher learning rate probably diverges from the planned output. It's projected to have the best learning rate in this environment. Optimization and reducing the neural network's loss function establish the learning rate. In the current experimental setup, the initial learning rate is considered 0.00001, and the models and the learning rate have saturated after the 26th epoch in the training process. The learning rate graph over the epochs for the model is shown in Fig. 9.

## Results and discussion

**Figure 10:**
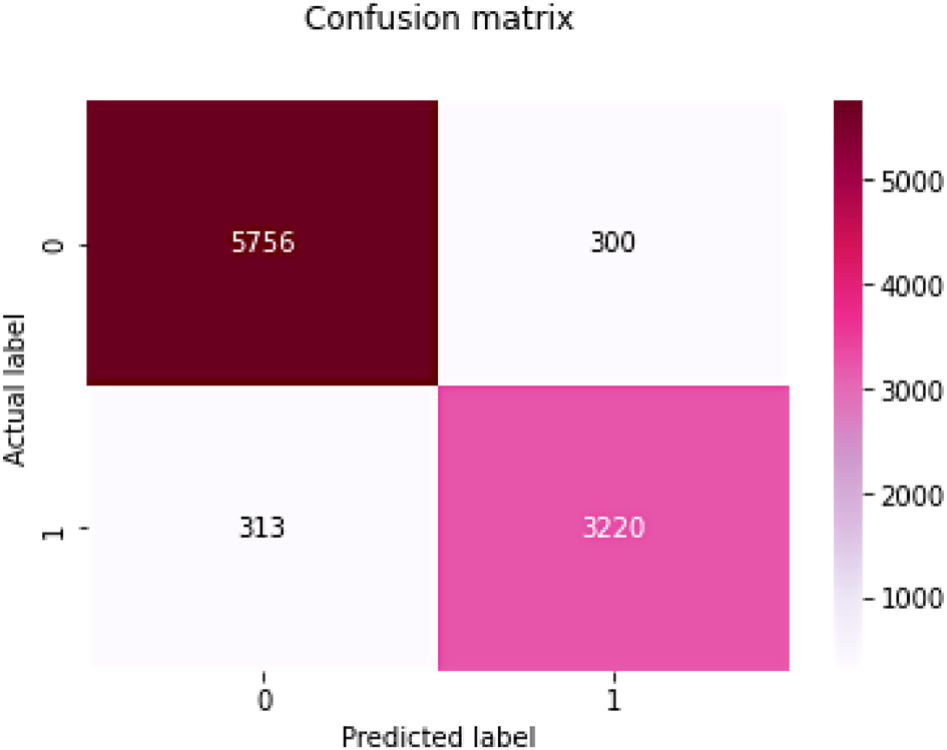
Confusing the matrix of ResNet-32 with FastAI.

The following part summarises the results of the suggested FastAI-driven ResNet-32 model's performance assessments. Performance is measured using sensitivity, specificity, Precision, Accuracy, and F1-Score parameters. The analysis is done following other cutting-edge models to evaluate the model's accuracy in categorizing ductal carcinoma tissue slides. When the model identifies a positive occurrence and the actual outcome is also positive, it is considered True Positive (Tpo). When the model identifies a negative event, and the actual result is negative, the outcome is True Negative (Tne). When the model detects an event as having a positive outcome, but the actual outcome is negative, the outcome is False Positive (Fpo). When the model identifies a negative outcome, but the actual outcome is positive, the outcome is False Negative (Fne)^45^. Tpo refers to properly identified tumors in a slide, while Tne refers to tumors that are mistakenly diagnosed in a slide. Figure 10 depicts the confusion matrix produced by the ResNet 32 model.

The confusion matrix is used in further evaluating the model concerning the measures like sensitivity which determines the ratio of the actual sum of true positive occurrences to the total sum of positive cases, and Specificity, which determines the ratio of the sum of true negative occurrences to the sum of genuine negative samples. Accuracy measures the proportion of participants whose labels are accurate to the total sum of actual positive occurrences considered. And similarly, F1-Score is used in approximating the class imbalance factor. The efficiency of various cutting-edge models concerning the evaluation parameters is shown in Table 6.

**Table 6:** Statistical analysis of various state-of-art models.

	Sensitivity (%)	Specificity (%)	Accuracy (%)	F1-Score (%)
Multi-layer perceptron^46^	76.0	74.0	76.0	–
Random Forest^28^	93.0	92.6	93.3	93.0
SVM^47^	90.0	95.50	92.75	–
SVM^48^	70.00	88.64	84.22	–
Random Forest^48^	90.44	70.30	75.55	–
Naïve Bayesian^48^	70.98	72.50	72.14	–
CNN^49^	84.70	–	84.93	76.07
AlexNet^50^	84.38	82.35	87.50	84.85
V66-16^51^	–	–	79.2	–
VGG-16^50^	83.24	81.29	86.36	83.74
VGG-19^50^	82.39	80.98	84.66	82.78
Inception V3 + SVM^51^	–	–	83.4	–
Inception V3^28^	80.5	82.0	79.0	81.0
Inception V3 + Bi-LSTM^51^	–	–	91.3	–
AlexNet^46^	93.6	91.7	92.7	–
AlexNet + SVM^52^	86.2	87.7	87.2	–
Faster RCNN (VGG)^53^	94.67	89.69	91.68	–
GoogLeNet^54^	91.70	97.66	91.70	91.92
ResNet-34^55^	89.37	81.79	90.66	84.19
VGG19^56^	91.16	97.66	91.16	91.18
Multiple interface learning-CNN^56^	94.43	77.78	88.81	–
Deep multiple instance learning-CNN^57^	94.44	88.89	93.06	–
Proposed model	94.83	91.48	93.60	95.90

**Figure 11:**
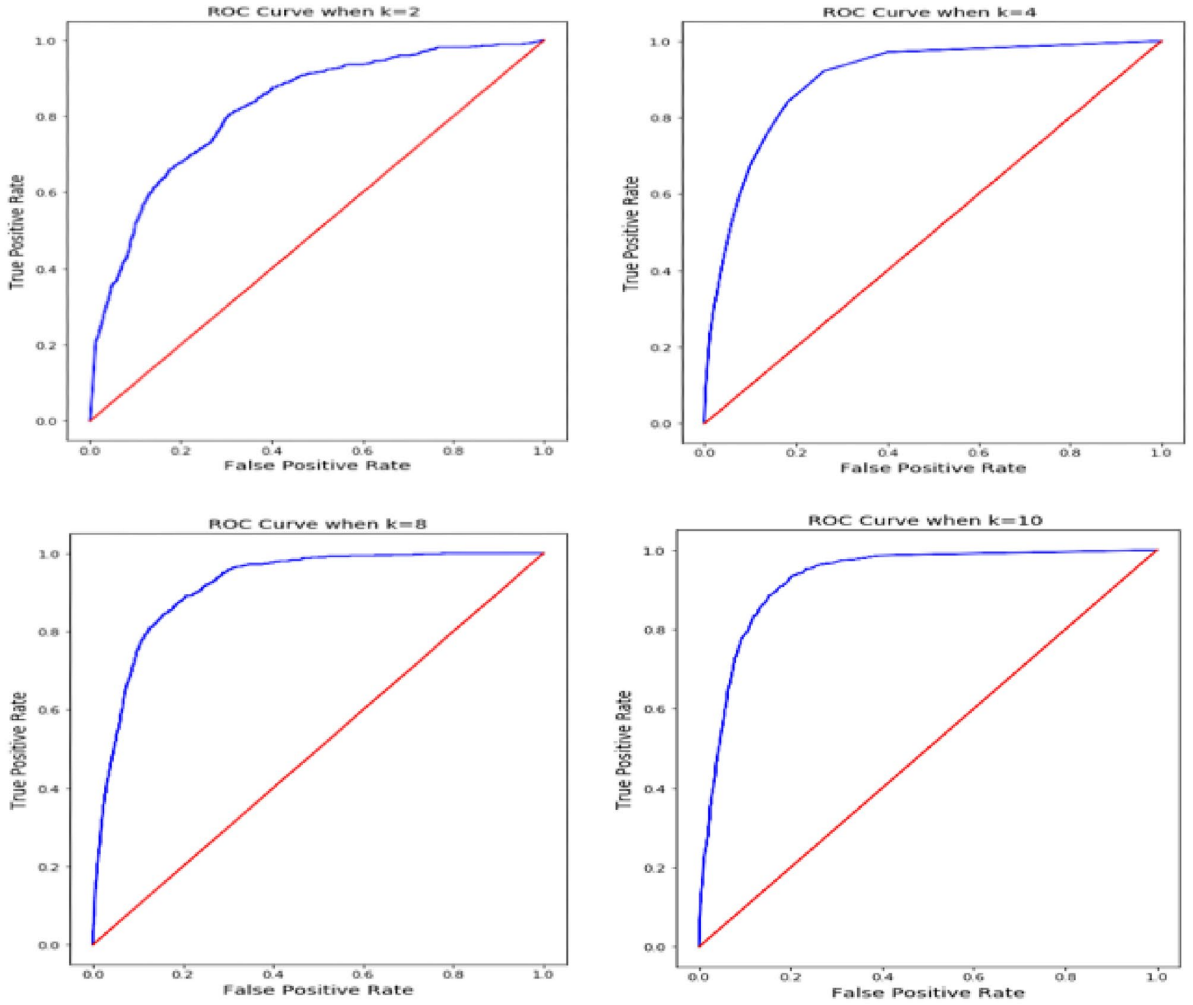
ROC curves associated with k-fold validation.

From the statistical analysis across various evaluation parameters shown in Table 6, the proposed FastAI-driven ResNet-32 model has surpassed in contrast to other existing models. The classification model has deliberated the outcome with a confidence of 98.6%, which is considered reasonable for a deep learning model. The model's performance is further evaluated using the Receiver Operating Characteristic curve (ROC), which is utilized to assess a classification model's efficiency. The ROC curves consider both the true and false positive rates for evaluating the model^58^. The ROC curve illustrates the association between sensitivity (or TPR) and specificity. Classifiers that generate lines nearer to the top-left corner function best. Cross-validation is another better model assessment approach than residuals. Residual assessments do not predict what the learner will perform when asked to generate new forecasts over the data that it has not previously seen. K subsets of the data are divided into k groups, and the holdout method is performed k times. An independent test set is performed for each group, with the remaining subsets of data being pooled to form a training set. For each k-trial, the average errors in those trials are determined. Figure 11 shows the ROC curves for the proposed model after it has been examined for a range of k-values.

It can be observed from the ROC curves across various The Diagnostic Odds Ratio (DOR)^59^ is a metric used in medical testing to evaluate the effectiveness of a certain diagnostic test for binary classification tasks. DOR is measured as the ratio of the chance of a positive test outcome if the patient has the illness to the probability of a positive test if indeed the individual does not have the disease, which is assessed as illustrated in Eq. (14)

14 $$DOR = \frac{Tpo \times Tne}{{Fpo \times Fne}}$$

The DOR of the current model is approximated as 196.75, which is a mean value of DOR considered for cross-validation for k as 2, 4, 8, and 10 in the experimentation process. The DOR values are assessed from the confusion matrix obtained on experimentation over the dataset, with 30% as the testing partition.

### Practical implications and future directions

Many methods have been developed over the last decade to aid in diagnosis and guide treatment decisions, but each method has its own set of advantages and disadvantages that prevent it from being used on its own. Recognizing the molecular diversity of a malignancy is essential for developing an effective therapeutic strategy. Different subgroups need different forms of systemic treatment and prognosis, making rapid categorization essential. Predictive and prognostic biomarkers from genomes, metabolic engineering, and proteomic of BC have formed the basis for a number of multigene tests that may be used to catch the disease early and tailor therapy accordingly. Because these multifunctional platforms may be utilised for the simultaneous identification, therapy, and monitoring of tumours, theranostics has emerged as a key tool in personalised medicine. Nanotheranostic formulations have unquestionable promise, but there are a number of considerations that must be made throughout development, testing, and implementation. The screening process and clinical outcomes community has a significant issue from the overdiagnosis of malignant tumours whose clinical relevance is uncertain. The screening process and clinical outcomes community has a significant issue from the overdiagnosis of malignant tumours whose clinical relevance is uncertain. More detailed image screening reports and disease progression will be available from future modelling work stratified by ductal carcinoma grade, genetic subtype, and intrinsic variables including breast cancer history in the family. There is great scope for research in the file of ductal carcinoma diagnosis by integrating the Electronic Healthcare Records (EHR), genomic sequences and imaging modularities would assist in precise diagnosis of the abnormality in the earlier stages.

## Concluding remarks

The current study on Ductal Carcinoma from the 2D Tissue Slides model is critical in personalized medicine because they help to identify high-risk people early on based on established disease-related risk factors. The proposed FastAI-driven ResNet-32 model has proven a comparable performance with faster computational efficiency. The study has shown that the models could learn complicated clinical information from images and distinguish tumor slides. The models were evaluated with divergent performance evaluation metrics. The model has proven to be more effective, with an accuracy of 93.6% on experimenting over the portion of the Invasive Ductal Carcinoma dataset. In the current study, the original size of tissue slide images is 50 × 50 and they are further downsampled to 32 × 32 size image for processing, that would result in missingout significant information from the images and that would compromise the performance of the model. The size of input image is also a concern for the applications in the healthcare and medical domains.

In the recent times in the healthcare domain, especially in cases of the diagnostic models the model, there is significant demand for the explainable models. Using Explainable Artificial Intelligence (XAI) techniques in feature selection and probability assessment in the classification process would make the decision making models interpretable. It is necessary to develop a self-learning model with feature engineering that would assist in working with the divergent feature set for precise classification of the abnormal slides. Moreover, the population size from where the slides are acquired is significantly important to training the model with divergent cases of abnormality. The study is limited to classification, and the survival analysis would further assist in analyzing the impact of the tumor growth on the individual.

## Data Availability

Dataset used in this research is publicly available at https://www.kaggle.com/datasets/paultimothymooney/breast-histopathology-images (accessed on 28 June 2022).
